# Modifications in Tissue and Cell Ultrastructure as Elements of Immunity-Like Reaction in *Chenopodium quinoa* against Prune Dwarf Virus (PDV)

**DOI:** 10.3390/cells9010148

**Published:** 2020-01-08

**Authors:** Edmund Kozieł, Katarzyna Otulak-Kozieł, Józef J. Bujarski

**Affiliations:** 1 Department of Botany, Institute of Biology, Warsaw University of Life Sciences-SGGW, Nowoursynowska Street 159, 02776 Warsaw, Poland; 2 Department of Biological Sciences, Northern Illinois University, DeKalb, IL 60115, USA; jbujarski@niu.edu

**Keywords:** prune dwarf virus, plant immunity, plant-virus interactions, virus systemic transport

## Abstract

Prune dwarf virus (PDV) is a plant RNA viral pathogen in many orchard trees worldwide. Our knowledge about resistance genes or resistant reactions of plant hosts to PDV is scant. To fill in part of this gap, an aim of this study was to investigate reactions to PDV infection in a model host, *Chenopodium quinoa*. Our investigations concentrated on morphological and ultrastructural changes after inoculation with PDV strain 0599. It turned out that PDV infection can cause deformations in host cells but also induce changes in the organelles, such as chloroplasts in inoculated leaves. Moreover, we also demonstrated specific reactions/changes, which could be associated with both types of vascular tissue capable of effectively blocking the systemic spread of PDV to upper leaves. Furthermore, the relative amount of virus, P1 protein deposition, and movement protein (MP) gene expression consequently decreased in PDV-inoculated leaves.

## 1. Introduction

Prune dwarf virus (PDV) is a viral pathogen distributed worldwide [1,2]. PDV, a member of the *Bromoviridae* family and the genus *Ilarvirus*, infects fruit trees, generates enormous decrease in fruit yield, and reduces the effectiveness of vegetative reproduction of various species of orchard trees [3]. For example, a decrease in fruit yield has been estimated at the level of 80–90% for sweet cherry [3,4,5]. Vegetative reproduction is crucial for orchard trees. A decrease during vegetative reproduction can reach up to 90% in nurseries [3,6]. PDV can also be transmitted via seeds and pollen of infected plants [3].

The PDV genome has a complex structure (Scheme S1) with three positive ssRNA segments, named RNA1, RNA2, and RNA3. RNA1 encodes P1 protein (replicase), which is the first element of the viral replication complex [1]. RNA2 encodes P2 protein, which serves as the RNA-dependent RNA polymerase (RdRp), a component of the replication complex [1,2]. The third segment, RNA3, encodes two proteins, coat protein (CP) and movement protein (MP). CP supports the “genome activation” process, which is crucial for replication of the viral RNA genome and also creates capsid for viral particles [7]. MP enables generation of tubular structures during cell-to-cell transport and supports translocation of viral particles via plasmodesmata [8].

In an infected host plant, PDV is transported both cell-to-cell and systemically throughout the whole plant. During local spreading (cell-to-cell movement), the virus is transported as viral particles via MP-generated tubular structures through plasmodesmata, which modify their size exclusion limit (SEL) [8]. In the case of systemic movement, our new data from infected tobacco and cucumber reveal that the transport is mainly associated with phloem and xylem cells [8,9,10].

There is very little knowledge about resistance genes or resistant reactions of plant hosts during PDV infection. Based upon Fulton’s research [11,12] and the Plant Viruses Online Database [13], we know that many species are incompatible with PDV infection and replication. These test plants are then potential resources for resistant reaction/genes for plant immunity. One of the most promising test plants is quinoa (*Chenopodium quinoa*). This plant is compatible with many members of the *Bromoviridae* family, for example, *Prunus necrotic ringspot virus* (PNRSV) of genus *Ilarvirus* [13]. Since PNRSV and PDV are similar viruses [2], the potential incompatibility of PDV may be due to a resistance reaction. Therefore, the aim of this study was to determine the reactions of quinoa to PDV inoculation in the context of ultrastructural changes that are associated with resistance. By using: PDV CP-targeted double antibody sandwich enzyme-linked immunosorbent assay (DAS-ELISA), immunofluorescence localization of viral P1 protein (the main component of the viral replication complex) and real-time quantitative polymerase chain reaction (qPCR) of PDV movement protein (MP) gene and localization of MP, the presence of PDV in inoculated leaves was confirmed. Moreover, by using microscopic methods, we demonstrated that changes in both types of vascular tissues are crucial for abolition of PDV systemic transport/spreading. Alterations in cell organelles were observed only in inoculated leaves, whereas DAS-ELISA, qPCR, and immunofluorescence excluded the presence of PDV in the stem and upper leaves. 

## 2. Materials and Methods

### 2.1. Virus Inoculation and DAS-ELISA 

Before electron transmission and light microcopy, DAS-ELISA was used on quinoa plants (*C. quinoa*). According to the Plant Virus Online database [13], this species is not susceptible to a broad spectrum of different isolates/strains of PDV. Fifty quinoa plants were mechanically inoculated as was presented in [9]. Mock- and PDV-inoculated leaves along with leaves and stems above were checked for the presence of PDV by using DAS-ELISA in 3 repeats for each time point, with primary antibodies against the PDV CP (Bioreba, Reinach, Switzerland), followed by purified anti-rabbit antibodies conjugated with alkaline phosphatase (Bioreba, Reinach, Switzerland) [14]. Each repeat was a new ELISA plate with samples. For each test, we took samples from 15 mock-inoculated and 15 PDV-inoculated plants. Readings of OD_405nm_ values were performed after 60 min. All DAS-ELISA tests were performed using the same reagents. The measurements were performed at 3 time intervals, 7, 14, and 20 dpi, for mock- and PDV-inoculated quinoa leaves and for systemic leaves. After 20 dpi, only systemic leaves were tested. The mean OD_405nm_ values from 3 DAS-ELISA tests were statistically assessed with one-factor analysis of variance (ANOVA) and evaluated at the *p* < 0.05 level of significance using Tukey’s post hoc honestly significant difference (HSD) test in Statistica software (version 13.0; StataSoft and TIBCO Software Inc., Palo Alto, CA, USA). For more precise assessment of DAS-ELISA results, we computed corrected mean OD_405nm_. To do this, we subtracted from the mean OD_405nm_ of sample PDV-inoculated plants a sum of mean OD_405nm_ of buffer and appropriate mock-inoculated plants. These data were also statistically assessed as above. As suggested by Paduch-Cichal et al. [15] and Paduch-Cichal and Sala-Rejczak [16], absorbance above 0.2 confirmed the presence of the virus. Significance threshold values of DAS-ELISA were determined according to [6,15,16]. Absorbance from mock-inoculated plants was much lower than this threshold.

### 2.2. Isolation of RNA and Genomic DNA (gDNA) and qPCR Analysis of Expression of MP Gene of PDV in Quinoa Plants

Parallel DAS-ELISA molecular analysis of *MP* gene expression based on qPCR was performed. This analysis was conducted with the same time intervals on a group of mock- and virus-inoculated plants as the DAS-ELISA test. Stem and leaf samples (the weight of each sample was 0.05 g) from mock- and virus-inoculated plants at the inoculation point and above (at 7, 14, and 20 dpi) were collected. From each plant we collected 6 leaf and 6 stem samples at each time point after inoculation. We repeated the whole experiment 3 times. During the experiment, we gathered samples from 90 mock- and 90 virus-inoculated plants. RNA from these samples was isolated by use of GeneMATRIX Universal RNA Purification Kit (EURx Sp. z o.o., Gdansk, Poland) according to the manufacturer’s protocol. From 6 selected samples, RNA and gDNA were isolated by use of GeneMATRIX Universal DNA/RNA/Protein Purification Kit (EURx Sp. z o.o., Gdansk, Poland) according to the manufacturer’s protocol. gDNA obtained in this way was used for preparation of calibration curves. In the next step, calibration curves were used to determine the efficiency of qPCR reaction for low-expression transcripts. Isolated RNA was purified from gDNA by on-column DNase I digestion (EURx Sp. z o.o., Gdansk, Poland). After, reaction samples were purified by use of GeneMATRIX Universal RNA Purification Kit (EURx Sp. z o.o., Gdansk, Poland) according to the manufacturer’s protocol. RNA concentration after purification was measured by NanoDrop 2000 (Thermo Fisher Scientific, Waltham, MA, USA). Quality of extracted RNA was checked with the use of electrophoresis in 1% agarose gel in denaturation conditions. Additional lack of RNA contamination was checked by RT-PCR reaction with *actin* (reference gene) primers on matrix of obtained RNA. This reaction showed that RNA did not have gDNA contamination. After contamination analysis, cDNA was obtained by use of NG dART RT kit (EURx Sp. z o.o., Gdansk, Poland) according to the manufacturer’s protocol. Reverse transcription reaction was performed in a volume of 10 µL (for reaction we used 700 ng of RNA formulation).

Reaction of qPCR was performed in a Bio-Rad CFX96 Touch^TM^ (Bio-Rad Poland Sp. z.o.o., Warsaw, Poland), using SsoAdvanced^TM^ Universal SYBR^®^ Green Supermix (Bio-Rad Polska Sp. z.o.o., Warsaw, Poland) with 2 already prepared 5-point calibration curves (based on cDNA and gDNA). Expression of PDV *MP* gene (gene ID: HM015770.1) in quinoa was investigated at different times after inoculation in different parts of the plant (stem and leaves). As reference gene, we used quinoa *actin* (gene ID: LOC110724665) in quinoa, as suggested in [17]. Primers for reference and investigated genes were designed in Primer3 software v. 0.4.0 (Primer3Plus, Free Software Foundation, Inc., Boston, MA, USA). Concentrations of primer sequences (for both genes) used for reaction and connection temperature are presented in Table S1. The starting solution of cDNA (used in preparation of calibration curves) was a 4× diluted mix of 12 randomly selected cDNA formulations. For calibration curves based on gDNA, it was a 10× diluted solution of gDNA. In all cases, other subsequent points in calibration curves were prepared by a series of 4× dilutions of mix. Reaction was performed in a volume of 15 µL, and 5 µL 10× diluted cDNA formulation of each analyzed gene was added. Conditions of qPCR reaction are presented in Table S2.

The most important parameters of RT-PCR reaction for all sequences are presented in Supplementary Table S3. Reaction efficiency and R^2^ factor, which define the quality of calibration curves for transcripts, remained within normal range (90–110%, >0.98, respectively). Analysis of melting curves indicated the presence of only one PCR product in each reaction. Level of expression of PDV *MP* in context (comparison) of expression of reference gene (quinoa actin) was calculated by use of the Gene Study tool in Bio-Rad CFX Connect software v. 1.1 (Bio-Rad Polska Sp. z.o.o., Warsaw, Poland). Statistical analysis of the results, which included calculation of relative gene expression levels and the significance of differences between tested samples (ANOVA method), was performed using the Gene Study tool. Results of this analysis were normalized using one reference gene encoding quinoa actin.

### 2.3. Immunofluorescence Localization of PDV-P1 in Quinoa Tissues and Assessment of Quantitative Fluorescence Signal

Two weeks after PDV inoculation, fragments of quinoa leaf blades that were analyzed with DAS-ELISA were treated, as previously described by Kozieł et al. [8]. To check the distribution of PDV, P1 protein was localized by immunofluorescence according to a procedure described by Kozieł et al. [9] with modification regarding secondary antibody. Purified rabbit polyclonal antibody anti-P1-PDV (GeneCust, Boynes, Luxemburg) served as primary antibody [9], while the secondary one was anti-rabbit antibody IgG with the attached AlexaFluor**^®^**488 (Abcam, Cambridge, UK). For better contrast of plant tissues, we added 4′,6-diamidine-2′-phenylindole dihydrochloride (DAPI). Slides were imaged on a PROVIS AX70 fluorescent microscope with Olympus UP90 high-definition camera (Olympus, Warsaw, Poland) using Olympus Cell Sense Standard Software (version 1.18; Olympus, Center Valley, PA, USA). The intensity of the green fluorescent signal from regions of localization of P1 was further analyzed by use of a quantitative measuring method, corrected total cell fluorescence (CTCF) [18]. For CTCF, 25 selected areas of every sample were analyzed. To measure the fluorescent signal levels, we used ImageJ (version 1.51k; National Institutes of Health., Bethesda, MD, USA). Measurements of the green immunofluorescence signal gained from ImageJ were calculated with CTCF at 20× magnification with 1.00 zoom factor by using the formula previously presented by Otulak-Kozieł et al. [18]:

Estimated CTCF values were then analyzed statistically at selected time intervals for all plants by using one-factor analysis of variance (ANOVA) as presented by [18]. 

### 2.4. Preparation of Leaf Material for Light and Transmission Microscopy 

Fragments of quinoa leaf tissue (at 7, 14, and 20 dpi) were cut out, fixed, and embedded with EPOXY resin exactly as described by Kozieł et al. [8], followed by slicing into thin sections for light microscope or ultrathin sections (100 nm) for TEM, and mounted on slides or copper grids, respectively [19]. Slides were stained with crystal violet, while ultrathin sections were contrasted with uranyl acetate/lead citrate (Sigma-Aldrich, St. Louis, MO, USA) for TEM.

### 2.5. Preparation of Leaf Material and Immunogold Localization of PDV Movement Protein (PDV MP) 

Fragments of quinoa leaf tissue was prepared according to Section 2.4. Immunogold localization was performed exactly according to the procedure presented by [8] with use of primary antibodies targeted PDV MP used previously in [8] and secondary anti mouse antibodies associated with 18 nm gold particles (JaksonImmunoResearch Europe, Cambridgeshire, UK).

### 2.6. Localization and Quantification of H_2_O_2_ by Corrected Total Electron Density (CTED) Method

Hydrogen peroxide was detected at selected time points by the method of Bestwick et al. [20] as modified by Otulak and Garbaczewska [21]. Briefly, quinoa leaf tissue was preincubated in 50 mM (*w*/*v*) 3-morpholinopropane-1-sulfonic acid (MOPS) buffer (pH 7.2) containing 5 mM CeCl_3_, washed for an hour with the same buffer, and fixed in 2% (*w*/*v*) paraformaldehyde/2% (*v*/*v*) glutaraldehyde in 0.05 M cacodylate buffer (pH 7.2–7.4) [21] for 2 h at room temperature. Samples were contrasted and fixed in 2% (*w*/*v*) OsO_4_ in cacodylate buffer and also dehydrated in a series of increasingly strong ethanol–water solutions as was presented by [21]. The material was gradually saturated with Epon 812 (Fluka) resin and polymerized for 24 h at 60 °C. Observations were made as previously described in [8]. To quantify the level of H_2_O_2_, negative photos were analyzed for distribution of electron-dense cerium (IV) perhydroxide precipitates by using the CTED method [22], with a general formula as follows:

CTED = Integrated Density − (Area of Selected Cell Region × Mean Density of Background Readings).

In the next step, CTED values were analyzed for statistical significance by using ANOVA). The mean CTED values were evaluated at the *p* < 0.05 level of significance using Tukey’s post hoc HSD test in Statistica software (version 13.0; StataSoft and TIBCO Software Inc., Palo Alto, CA, USA).

### 2.7. Measurment of H_2_O_2_ Concentration

The hydrogen peroxide (H_2_O_2_) levels in mock- and PDV-inoculated quinoa leaves and leaves above the PDV inoculation point were determined according to Velikova et al. [23] at 7 and 14 dpi. Frozen leaves (0.5 g) were extracted in an ice bath with 5 mL 0.1% (*w*/*v*) tri-chloro-acetic acid (TCA). The homogenate was centrifuged at 15,000× *g* for 15 min. Then, an aliquot (0.5 mL) of the supernatant was added to a mixture of 0.5 mL potassium phosphate buffer (10 mM, pH 7.0) and 1 mL 1 M KI. After 20 min of incubation in darkness at room temperature, the absorbance of the samples was spectrophotometrically determined at 390 nm [23] by use of a SmartSpec™ 3000 spectrophotometer (Bio-Rad, Philadelphia, PA, USA). The concentration of H_2_O_2_ was calculated from a standard curve.

## 3. Results

### 3.1. Symptoms of PDV Infection and Immunofluorescent Localization of P1 Protein in a Context of PDV CP Distribution and Relative Expression of MP

**Figure 1:**
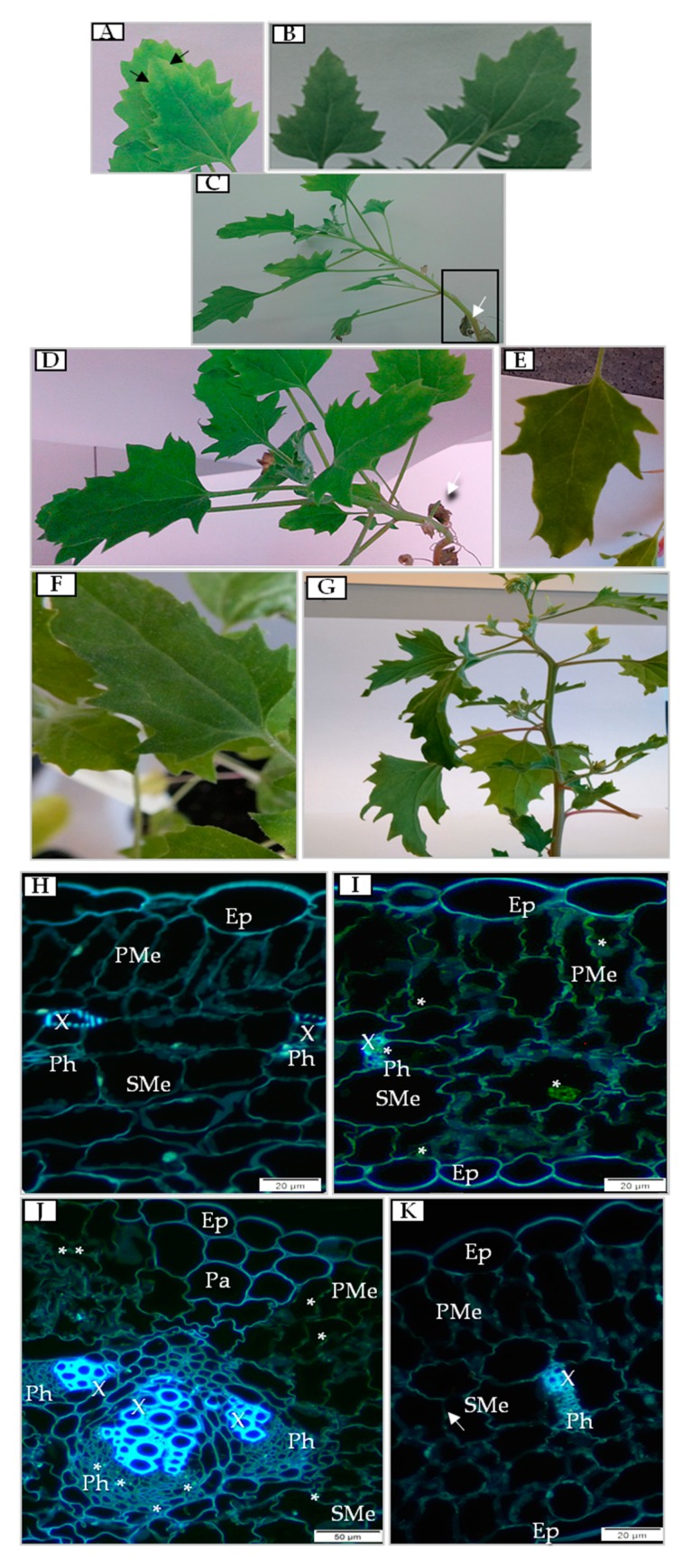
Symptoms of prune dwarf virus (PDV) infection on quinoa leaves and cellular deposition of PDV P1 protein. (**A**) Symptoms of PDV infection (black arrows) at 7 days post-inoculation (dpi). (**B**) Mock-inoculated quinoa leaves at 7 dpi. (**C**) Necrotization of PDV-inoculated quinoa leaves at 14 dpi (white arrow). Black framed area is presented in (**C**). (**D**) Altered leaf of PDV-inoculated quinoa at 14 dpi (white arrow). (**E**) Mock-inoculated quinoa plant leaf at 14 dpi. (**F**) Upper leaf of PDV-inoculated plant at 14 dpi. (**G**) Lack of systemic symptoms on leaves after virus eradication at 20 dpi. (**H**) Mock-inoculated quinoa leaves at 7 dpi. (**I**) Immunofluorescent visualization of P1 in inoculated leaves at 7 dpi (green fluorescence, marked with *). (**J**) Deposition of P1 at 14 dpi (green fluorescence, marked with *). Deformation of palisade and spongy mesophyll cells is marked with white arrow. (**K**) Absence of P1 in upper leaves at 20 dpi. Abbreviations: Ep, epidermis; PMe, palisade mesophyll; SMe, spongy mesophyll; X, xylem; Ph, phloem; Pa, parenchyma.

PDV first caused symptoms approximately 7 days post-inoculation (dpi) on inoculated leaves, and chlorotic lesions developed near the edges of leaf blades (Figure 1A), which did not occur on mock-inoculated plants (Figure 1B). After 14 dpi, the inoculated leaves became necrotized (Figure 1C,D), but mock-inoculated leaves (Figure 1E) and upper leaves of PDV-inoculated plants were unchanged and had no symptoms of infection (Figure 1F). The inoculated necrotized leaves got detached from the plant about 20 dpi (Figure 1G). To check whether the observed symptoms were caused by ongoing PDV infection, P1 protein was identified/localized by immunofluorescence; the presence of P1 was not detected in mock-inoculated leaves after 7 dpi (Figure 1H). Green fluorescent signal of PDV P1 protein was spotted starting at 7 dpi inside both palisade and spongy mesophyll, but not in phloem and xylem tissue (Figure 1I). At 14 dpi, the P1 fluorescent signal was less intense than at 7 dpi. P1 protein deposition was detected not only in both types of mesophyll but also in the phloem (Figure 1J). Moreover, numerous palisade and spongy mesophyll cells were deformed (Figure 1J). Such alterations were clearly visible as cell wall invaginations (Figure 1J). In contrast to PDV-inoculated leaves at 14 dpi, P1 was not detectable in upper leaves even at 20 dpi (Figure 1K). Such deposition patterns suggest that PDV replication inside quinoa plants is associated with and limited to inoculated leaves.

**Figure 2:**
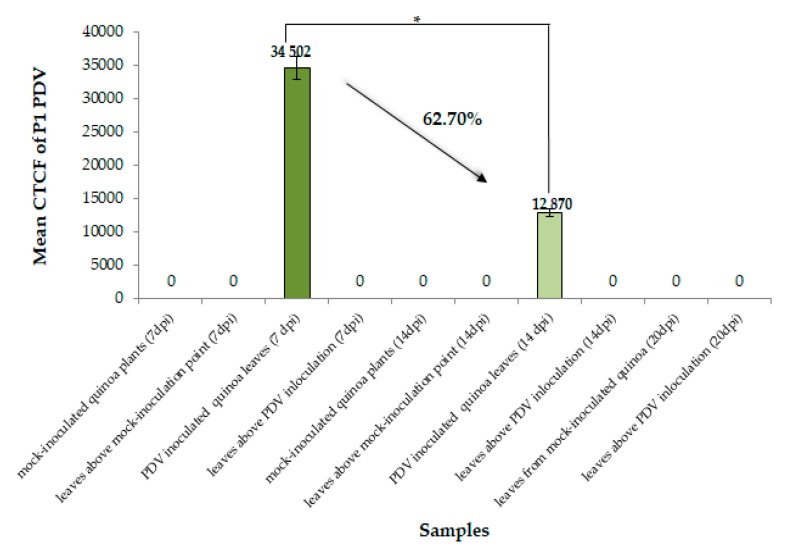
Quantitative fluorescence signals of P1 protein of PDV using corrected total cell fluorescence method (CTCF) combined with ANOVA statistics analysis at 7 and 14 dpi. Black arrow indicates % decrease of CTFC value. Mean CTCF values were evaluated at the *p* < 0.05 level of significance using Tukey’s post hoc honestly significant difference (HSD) test. Black bracket with asterisk (*) indicates significant statistical difference between PDV-inoculated quinoa leaves at 7 and 14 dpi.

Moreover, quantitative measurement of the fluorescence signal based on corrected total cell fluorescence (CTCF) confirmed a statistically significant decrease of P1 protein signal (approximately 62%) between 7 and 14 dpi and the absence of P1 in upper leaves of PDV-inoculated plants (Figure 2).

To quantify relative virus concentration, DAS-ELISA was performed by using anti-PDV CP antibodies. This test confirmed the presence of PDV in the inoculated leaves at 7 and 14 dpi (Table S4, Figure S1A,B), but PDV was not detected in upper leaves or stems at 14 and 20 dpi (Figure S1). Moreover, statistical analysis of the optical density (OD_405_) and corrected optical density (corrected OD_405_) data from DAS-ELISA confirmed a significantly decreased relative concentration of CP/virus by 55.36% (mean OD_405_) or 56.74% (corrected OD_405_) between 7 and 14 dpi time points (Figure S1A,B).

**Figure 3:**
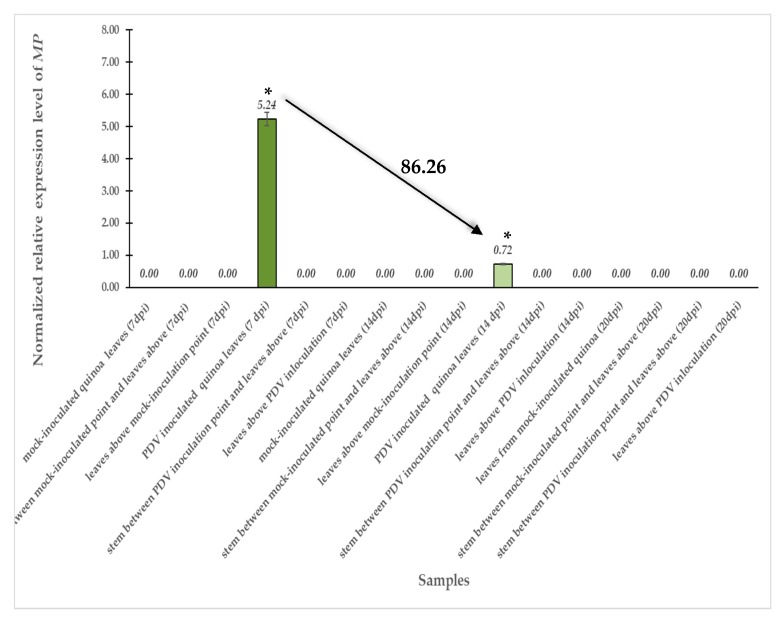
Normalized relative expression level of *MP* in mock- and virus-inoculated leaves and stem above inoculation point combined with ANOVA at 7, 14, and 20 dpi. Mean values of normalized expression were evaluated at the *p* < 0.05 level of significance using Tukey’s post hoc HSD test. Statistically significant values are marked by asterisks (*) above mean values of normalized expressions on each bar.

Furthermore, real-time quantitative polymerase chain reaction (qPCR) expression analysis of PDV movement protein was performed. Statistical analysis of qPCR results revealed that PDV *MP* gene was expressed only in virus-inoculated quinoa leaves at 7 and 14 dpi (Figure 3). Normalized relative expression level of *MP* gene statistically significantly decreased (approximately 86%) in inoculated leaves between 7 and 14 dpi. Moreover, in stem and leaves above the inoculation point, no expression was observed at 7, 14, and 20 dpi, as well as in all parts of mock-inoculated plants (Figure 3).

### 3.2. Anatomical and Ultrastructural Changes in PDV-Infected Quinoa Plants and Immunogold Labbleing of PDV MP

**Figure 4:**
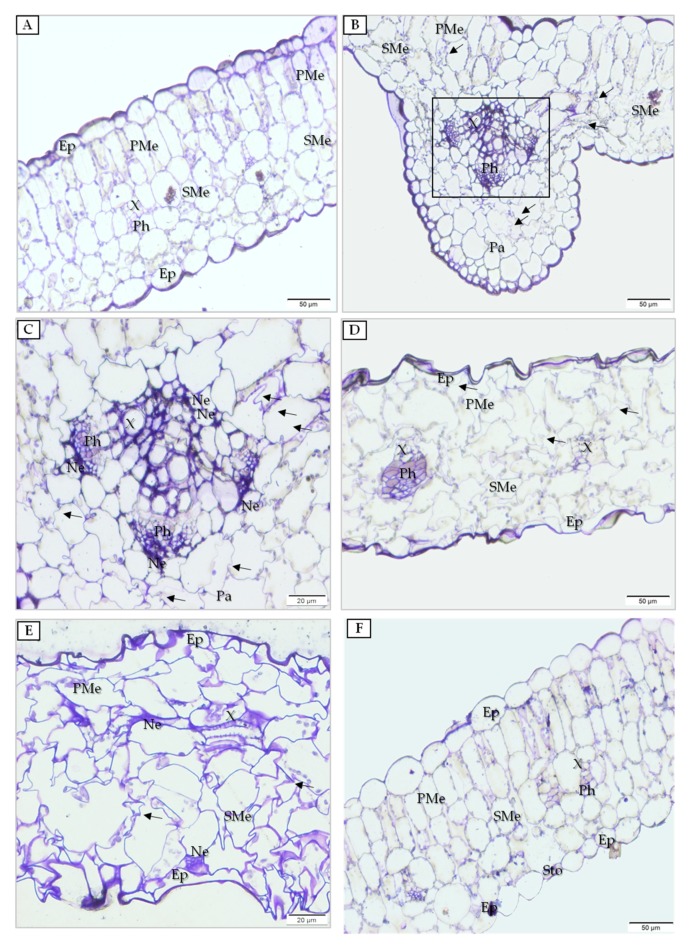
Anatomical alterations induced by PDV in quinoa leaves. (**A**) Mock-inoculated quinoa leaf; bar, 50 µm. (**B**) Inoculated leaf with deformations (black arrows) at 14 dpi. Black framed area presented in (**C**); bar, 50 µm. (**C**) Changes in parenchyma cell wall (black arrows) and in phloem and xylem parenchyma of major vascular bundle at 14 dpi; bar, 20 µm. (**D**) Fragment of leaf with distant vascular bundle. Numerous deformations (black arrows); bar, 50 µm. (**E**) Inoculated leaf with deformations (black arrows) at 20 dpi combined with local necrotic alterations near xylem and spongy mesophyll; bar, 50 µm. (**F**) No anatomical alterations at 14 dpi in upper leaf; bar, 20 µm. Abbreviations: Ep, epidermis; PMe, palisade mesophyll; SMe, spongy mesophyll; X, xylem; Ph, phloem; Ne, necrosis; Pa, parenchyma; Sto, stomata.

Mock-inoculated quinoa plants (at 7 dpi) displayed unaffected cells in all tissues of the leaf (Figure 4A). Anatomical changes were observed only in the inoculated leaves (Figure 4B). In this case, high levels of deformation were observed in spongy mesophyll and in parenchyma cells (14 dpi) (Figure 4B). At 14 dpi, we also spotted invagination of cell wall into this tissue (Figure 4B,C) as well as necrosis in both phloem and xylem parenchyma inside major vascular bundles (Figure 4C). More severe changes were observed in PDV-inoculated leaves at some distance from major vascular bundles at 14 dpi in the form of numerous deformations and cell-wall invaginations, not only in spongy mesophyll but also in epidermis and palisade mesophyll (Figure 4D). In PDV-inoculated leaves (20 dpi), the level of deformations and alterations related to local necrotization was increased in both xylem and spongy mesophyll (Figure 4E). Such anatomical alterations and cell deformations were not observed at 14 dpi in upper non-inoculated leaves (Figure 4F).

**Figure 5:**
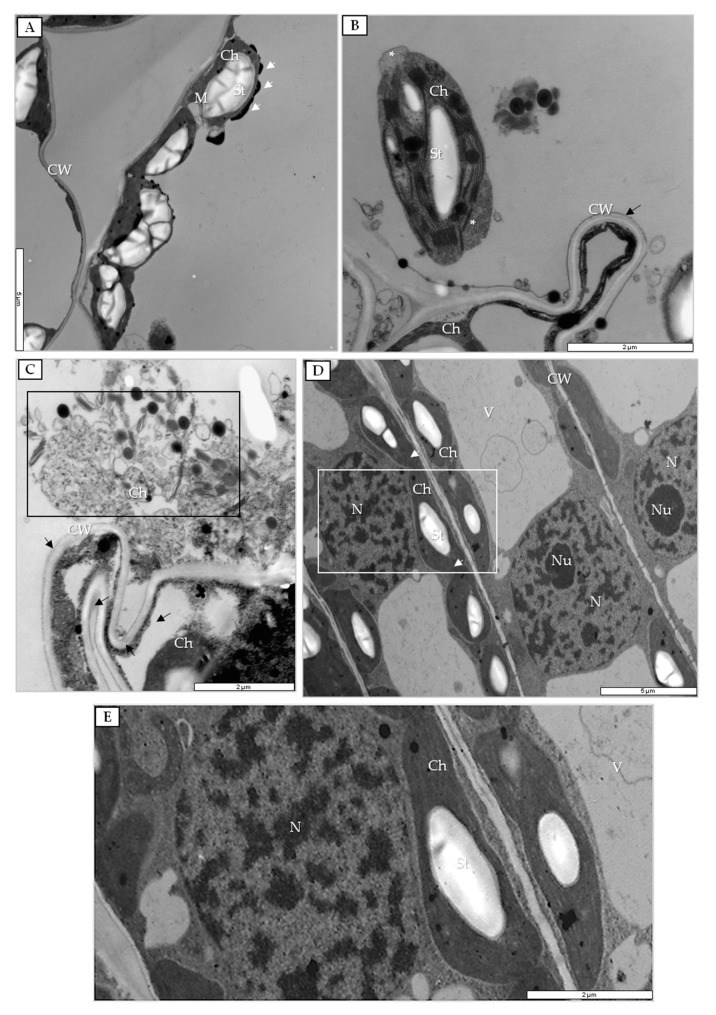
Ultrastructure of chloroplasts in PDV- and mock-inoculated quinoa leaves. (**A**) Palisade mesophyll cell at 7 dpi with chloroplast coated by electron-dense substance (white arrows); bar, 5 µm. (**B**) Changes of chloroplast envelope and cell wall alteration in spongy parenchyma cells at 14 dpi; bar, 2 µm. (**C**) Disintegrated chloroplast (black frame) at 20 dpi; bar 2 µm. (**D**) Palisade parenchyma with chloroplasts (white arrows) in mock-inoculated quinoa leaves at 14 dpi; bar, 5 µm. White-framed area enlarged in (**E**); bar, 2 µm. (**E**) Chloroplasts from mock-inoculated quinoa leaves at 14 dpi; bar, 2 µm. Abbreviations: CW, cell wall; Ch, chloroplast; N, nucleus; Nu, nucleolus; M, mitochondrion; St, starch.

For deeper analysis of the course of PDV infection at the ultrastructural level, the tissues were examined by transmission electron microscopy (TEM). Changes were first observed in chloroplasts in PDV-inoculated leaves at 7 dpi (Figure 5A). Many chloroplasts in palisade mesophyll cells accumulated electron-dense substances (lipids). Later, alterations in chloroplasts became more intense, with spotted invaginations of chloroplast envelope at 14 dpi (Figure 5B); likewise, invaginations of cell wall in spongy mesophyll cells were spotted. At 20 dpi, many disintegrated chloroplasts and deep invaginations of cell wall could be found (Figure 5C). In comparison, in mock-inoculated plants, changes in chloroplasts did not occur (Figure 5D,E).

**Figure 6:**
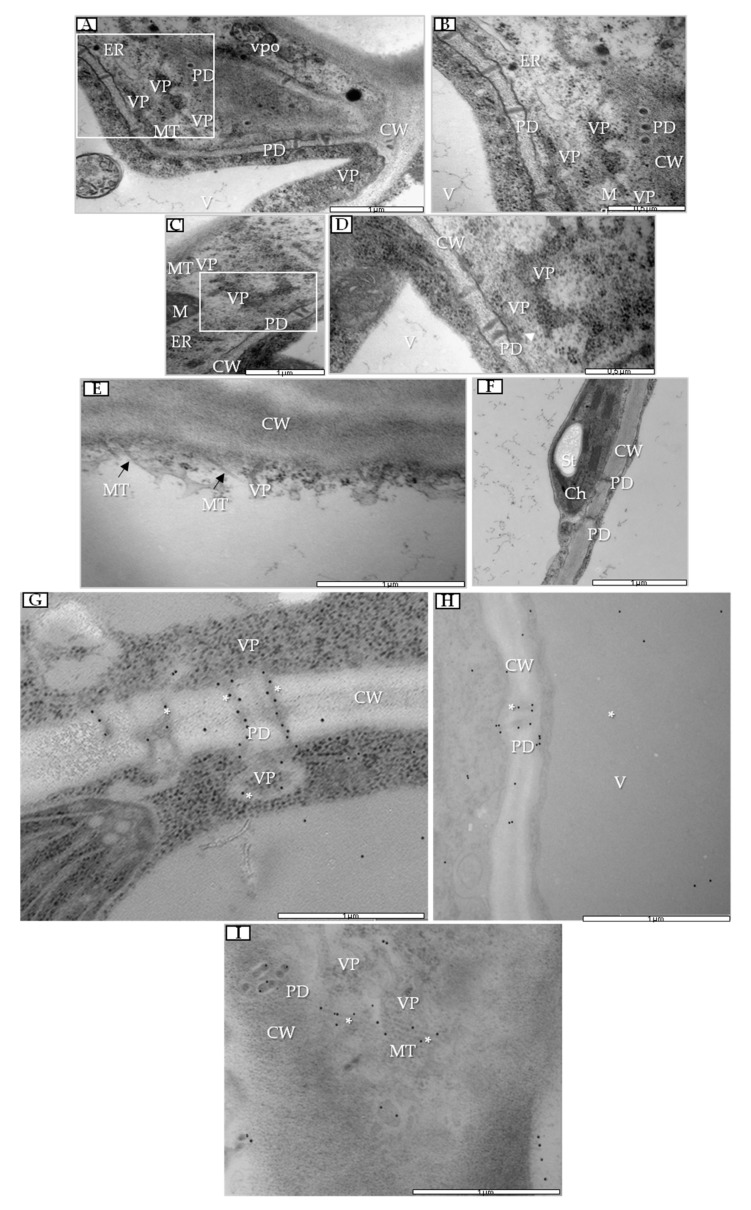
Changes in parenchyma cells in PDV-inoculated quinoa leaf and immunogold labeling of PDV MP in parenchyma cells. (**A**) Parenchyma cell at 7 dpi with PDV viral particles (VPs). White-framed area enlarged in (**B**); bar, 1 µm. (**B**) Virus particles at 7 dpi; bar, 0.5 µm. (**C**) Parenchyma cell of PDV-infected quinoa at 14 dpi. White-framed area enlarged in (**D**); bar, 1 µm. (**D**) PDV VPs inside extended plasmodesmata (white arrow) at 14 dpi; bar, 0.5 µm. (**E**) PDV VP near potential MP-induced tubular structures passing through parenchyma cell wall; bar, 1 µm. (**F**) Parenchyma cell from mock-inoculated plant at 14 dpi; bar, 1 µm. (**G**) PDV MP epitopes (*) inside plasmodesmata at 14 dpi; bar, 1 µm. (**H**) PDV MP epitopes (*) inside extended plasmodesmata at 14 dpi; bar, 1 µm. (**I**) PDV MP epitopes (*) inside potential MP-induced tubular structures passing through parenchyma cell wall near plasmodesmata at 14 dpi; bar, 1 µm. Abbreviations: CW, cell wall; PD, plasmodesmata; MT (with black arrow), movement protein-induced tubular structure; ER, endoplasmic reticulum; VP, viral particle; V, vacuole; vpo, vesicle pocket; M, mitochondrion.

During infection, especially at 7 dpi, we observed the presence of PDV viral particles (VPs) in parenchyma cells (Figure 6A,B) alongside vesicle pockets (Figure 6A) and enlarged endoplasmic reticulum (ER) cisterns (Figure 6B). PDV VPs were also often observed near plasmodesmata, which had an extended size exclusion limit (SEL) at 14 dpi, which thus enabled PDV cell-to-cell transport (Figure 6C,D). More importantly, PDV VPs were found near potential movement protein-induced tubular structures (MTs) that passed through to the cell wall (Figure 6E), which was not observed in mock-inoculated parenchyma cells (Figure 6F). To check potential association of plasmodesmata and MT changes, immunogold labeling of PDV MP was performed. During infection we observed presence of PDV MP epitopes inside plasmodesmata (Figure 6G,H) and also inside potential MTs in parenchyma cells (Figure 6I).

**Figure 7:**
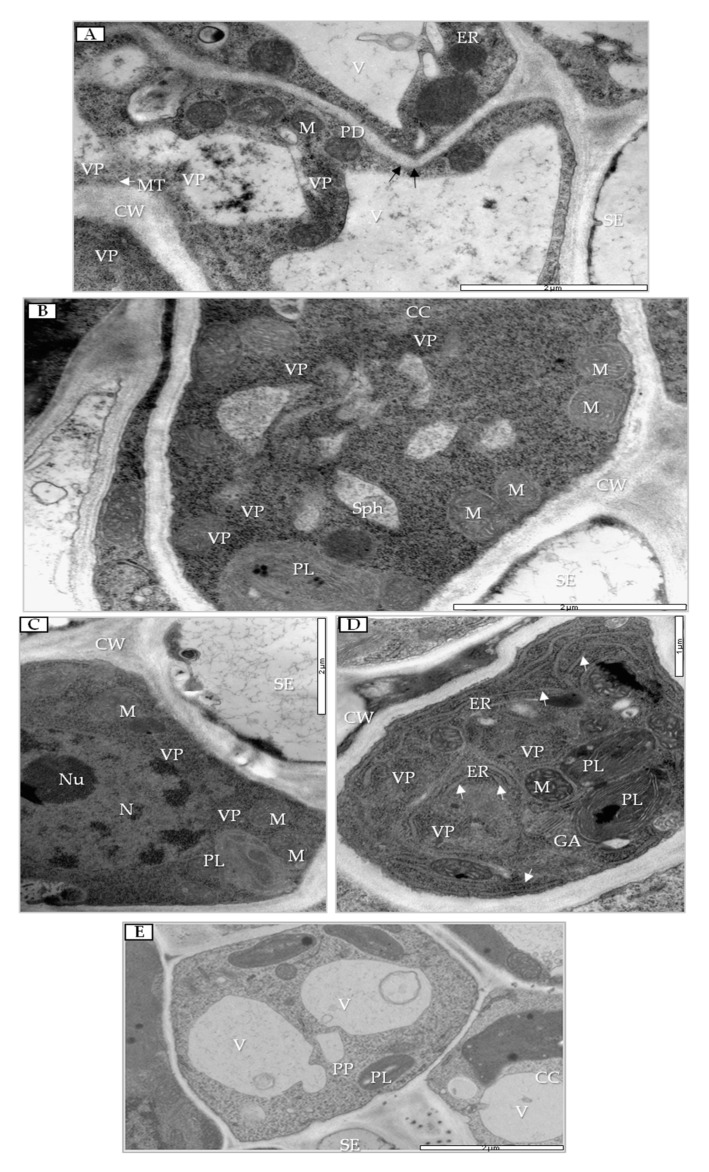
Changes in phloem of PDV-inoculated quinoa leaves. (**A**) Viral particles (VPs) and cell wall invagination (black arrows) at 14 dpi. VPs near tubular structures in cell wall of parenchyma (white arrow); bar, 2 µm. (**B**) Companion cell with viral particles and spherules at 14 dpi; bar, 2 µm. (**C**) Viral particles in companion cell; bar, 2 µm. (**D**) Enlarged endoplasmic reticulum cisterns (white arrows) and Golgi apparatus at 14 dpi; bar 1 µm. (**E**) Phloem of mock-inoculated quinoa plant at 14 dpi. Abbreviations: CW, cell wall; V, vacuole; ER, endoplasmic reticulum; VP, viral particle; Sph, spherule; M, mitochondrion; CC, companion cell; SE, sieve tube; PL, plastid. N, nucleus; Nu, nucleolus; GA, Golgi apparatus.

In PDV-inoculated leaves at 14 dpi, viral particles were present in phloem parenchyma and companion cells (Figure 7A,B). In phloem parenchyma, the nearby sieve tube cell wall was often dented and had tubular structures accompanied by PDV VPs (Figure 7A). In companion cells, the observed changes at 14 dpi concerned high accumulation of viral particles and formation of spherules in vacuoles (Figure 7B). Moreover, VPs were mostly present near the nucleus (Figure 7C) in companion cells, followed by enlarged endoplasmic reticulum (ER) cisterns and Golgi apparatus (Figure 7D). Spherules and VPs were not noticed in cells from mock-inoculated tissues (Figure 7E).

**Figure 8:**
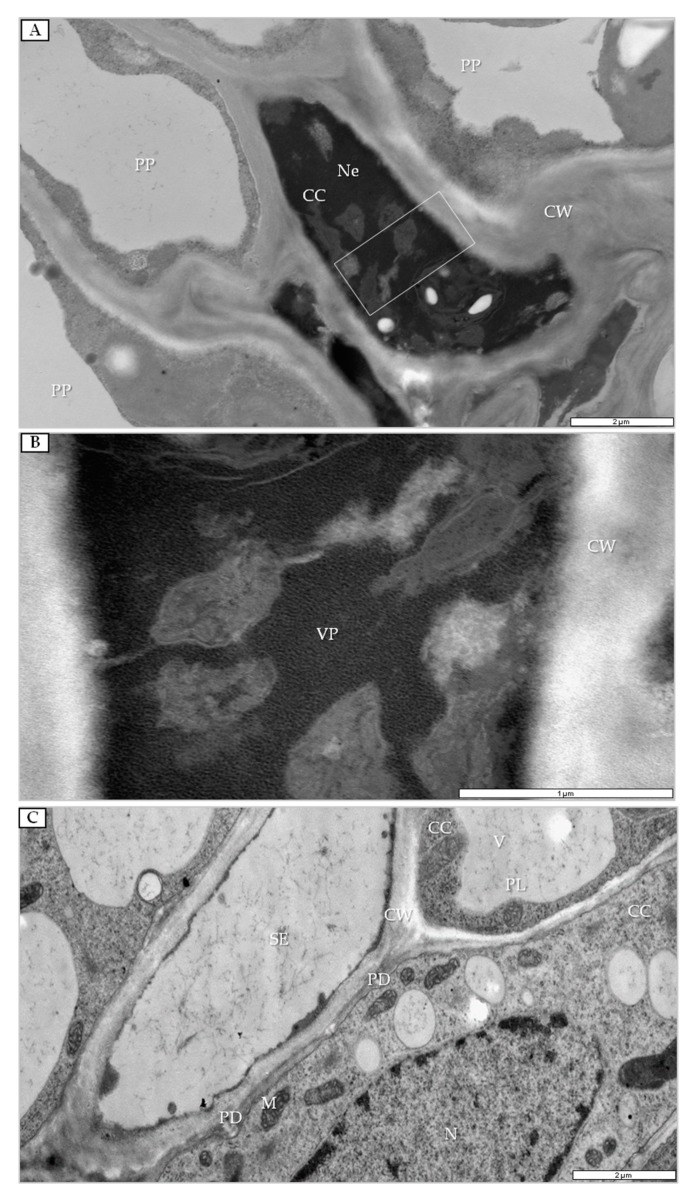
Companion cells of PDV- and mock-inoculated quinoa leaves at 14 dpi. (**A**) Changed companion cell. White frame enlarged in (**B**); bar, 2 µm. (**B**) Changed companion cell with multiple viral particles; bar, 1 µm. (**C**) Companion cells in mock-inoculated quinoa leaf; bar, 2 µm. Abbreviations: CC, companion cell; PP, phloem parenchyma; N, nucleus; PL, plastid; SE, sieve tube; V, vacuole; Ne. necrosis; CW, cell wall; VP, viral particle.

**Figure 9:**
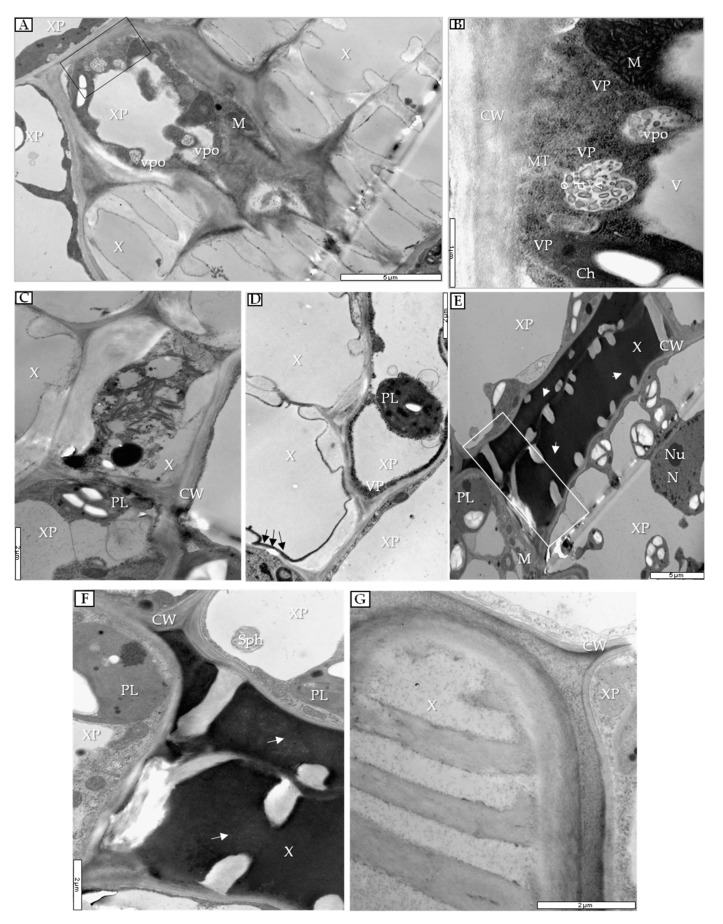
Xylem parenchyma in PDV- and mock-inoculated quinoa leaf at 14 dpi and also xylem tracheary elements in quinoa leaf at 7, 14, and 20 dpi. (**A**) Vesicle pockets in xylem parenchyma cell. Black frame enlarged in (**B**); bar, 5 µm. (**B**) VPs and tubular structures inside xylem parenchyma cell; bar, 1 µm. (**C**) Developing xylem tracheary element at 7 dpi; bar, 2 µm. (**D**) Electron-dense layer along cell wall (black arrows), xylem parenchyma cell containing VPs at 14 dpi; bar, 2 µm. (**E**) Electron-dense substance (white arrows) in whole xylem tracheary element at 20 dpi. White frame enlarged in (**F**); bar, 5 µm. (**F**) Electron-dense substance (white arrows) in xylem tracheary element and spherule in xylem parenchyma cell; bar, 2 µm. (**G**) Xylem from mock-inoculated quinoa plant at 20 dpi; bar, 2 µm. Abbreviations: X, xylem vessel; XP, xylem parenchyma; CW, cell wall; PL, plastid; VP, viral particle; Sph, spherule; N, nucleus; Nu, nucleolus; M, mitochondrion; vpo, vesicle pocket; Ch, chloroplast; V, vacuole;; MT -movement protein-induced tubular structure.

At 14 dpi, necrotic changes occurred in phloem companion cells (Figure 8A) concomitant with the presence of viral particles (Figure 8B). In companion cells of mock-inoculated plants, the presence of PDV was not noticed (Figure 8C). Moreover, changes were noticed in xylem tissue, whereas viral particles were detected only in xylem parenchyma (Figure 9A,B) with vesicle pockets or near tubular structures (Figure 9B).

Furthermore, reactions were also observed in xylem tracheary elements, accumulating electron-dense material (probably phenols), starting from 7 dpi (Figure 9C). Such electron-dense material could be observed first along internal parts of cell walls (Figure 9D). Finally, at 20 dpi this electron-dense substance filled the xylem tracheary elements completely (Figure 9E,F). Electron-dense material did not accumulate in mock-inoculated xylem (Figure 9G).

**Figure 10:**
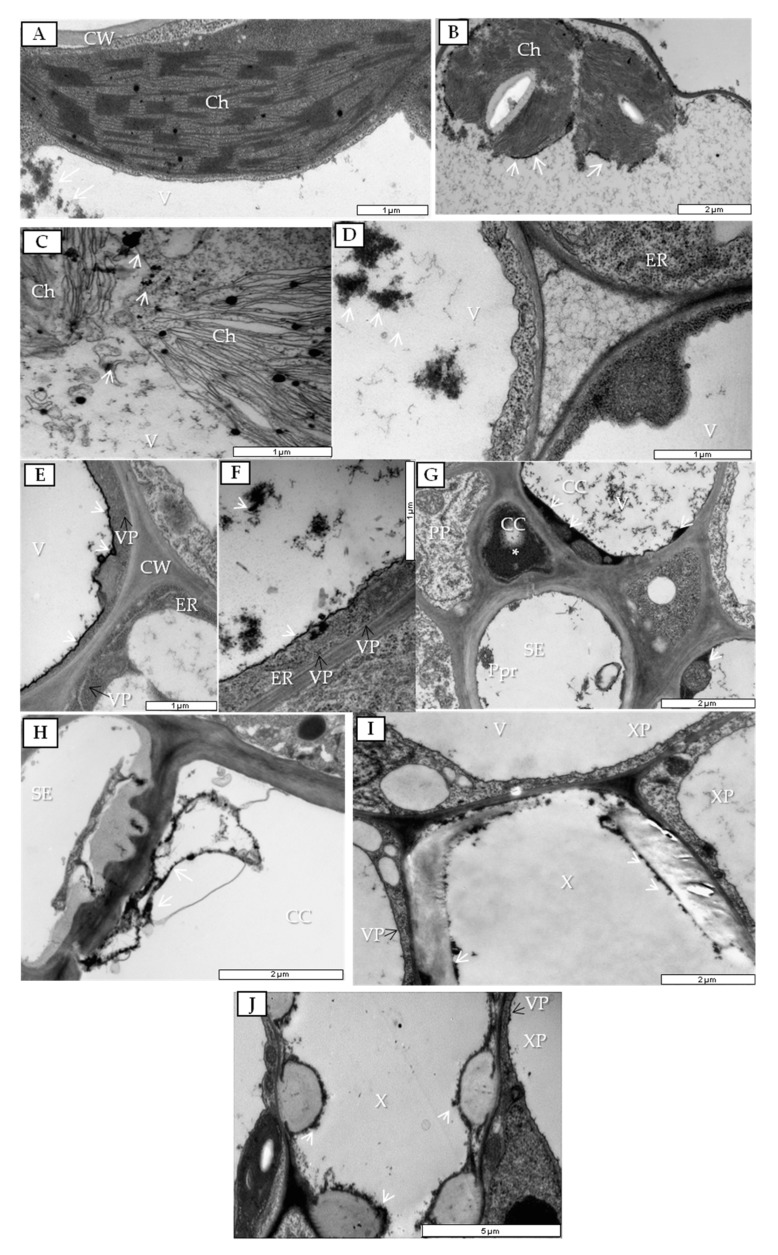
Localization of H_2_O_2_ in mock- and PDV-inoculated quinoa leaves. (**A**) Deposition of H_2_O_2_ (white arrow) in vacuole of palisade mesophyll cell from mock-inoculated leaf; bar, 1 µm. (**B**) H_2_O_2_ layer (white arrow) near chloroplast envelope at 7 dpi in mesophyll cell of inoculated leaf; bar, 2 µm. (**C**) Deposition of H_2_O_2_ (white arrows) near disintegrated chloroplasts in mesophyll cell of inoculated leaf at 14 dpi; bar, 1 µm. (**D**) H_2_O_2_ in a vacuole of spongy mesophyll cell (white arrows). (**E**) H_2_O_2_ along vacuole tonoplast (white arrows) inside companion cell with PDV particles at 14 dpi (black arrows); bar, 1 µm. (**F**) H_2_O_2_ (white arrows) along vacuole tonoplast and inside the vacuole of companion cell with PDV particles at 14 dpi; bar, 1 µm. (**G**) Necrotization of companion cell (*) and H_2_O_2_ (white arrows) in companion cells at 20 dpi; bar, 2 µm. (**H**) H_2_O_2_ (white arrows) in necrotic companion cell at 20 dpi; bar, 2 µm. (**I**) H_2_O_2_ (white arrows) along cell wall of xylem tracheary element at 14 dpi. VPs (black arrows) in xylem parenchyma. H_2_O_2_ in xylem tracheary element; bar, 2 µm. (**J**) H_2_O_2_ in xylem tracheary element at 20 dpi; bar, 5 µm. Abbreviations: Ch, chloroplast; ER, endoplasmic reticulum; V, vacuole; CW, cell wall; VP, viral particle; PP, phloem parenchyma; CC, companion cell; SE, sieve tube; Ppr, phloem protein; XP, xylem parenchyma; X, xylem vessel.

To define the source of ultrastructural changes, the tissue was tested for hydrogen peroxide (H_2_O_2_) because reactive oxygen species (ROS) may induce the above alterations during transduction of the infection signal. Detection of H_2_O_2_ in quinoa cells was accomplished by using CeCl_3_, which reacts with H_2_O_2_, generating an electron-dense cerium (IV) perhydroxide precipitate. For mock-inoculated plants, deposition of such precipitate was observed rarely and in small amounts in vacuoles (Figure 10A). However, precipitates with H_2_O_2_ were spotted as layers on the surface of altered chloroplasts in the mesophyll at 7 dpi (Figure 10B). At 14 dpi, the presence of H_2_O_2_ was confirmed near disintegrated chloroplasts (Figure 10C) and at high levels in mesophyll vacuoles (Figure 10D). Moreover, these depositions were also related to changes in cells of both types in vascular tissues. High accumulation of H_2_O_2_ was first observed in companion cells at 14 dpi as an electron-dense layer along the tonoplast near viral particles in cytoplasm (Figure 10E,F). Later on, H_2_O_2_ was not only in tonoplast but also in necrotically changed cytoplasm (Figure 10G) or in disintegrated cytoplasm of companion cells at 20 dpi (Figure 10H). Furthermore, in xylem we observed H_2_O_2_ in tracheary element at 14 dpi (Figure 10I), and the level of H_2_O_2_ increased with time at 20 dpi (Figure 10J).

The distribution of H_2_O_2_ was quantified statistically based on the corrected total electron density (CTED) tool (Figure S2) by measuring the density of cerium (IV) perhydroxide precipitate. In mock-inoculated plants from 7 to 14 dpi, CTED slightly increased but not to a statistically significant level (Figure S2), whereas the level of H_2_O_2_ was higher in PDV-inoculated leaves than in mock-inoculated plants, increasing with time between 7 and 14 dpi (Figure S2) to over 92%.

**Figure 11:**
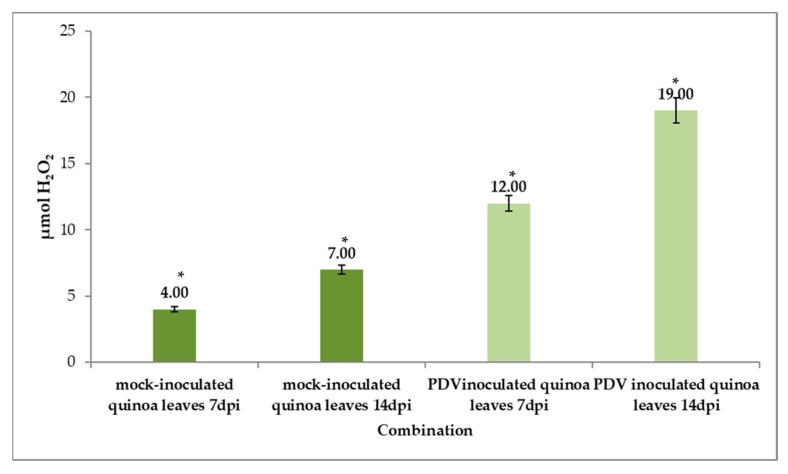
H_2_O_2_ concentration in mock- and PDV-inoculated quinoa leaves (7 and 14 dpi), assessed in combination with ANOVA statistics. Mean values of H_2_O_2_ concentration were evaluated at the *p* < 0.05 level of significance using Tukey’s post hoc HSD test. Significant values (*).

CTED results were also confirmed by spectrophotometric analysis of H_2_O_2_ concentration in mock- and PDV-inoculated leaves. These data clearly indicated that PDV inoculation significantly increased H_2_O_2_ levels at 7 and 14 dpi (from 12 to 19 µmol H_2_O_2_) compared to mock-inoculated quinoa plants (Figure 11. Moreover, H_2_O_2_ concentration was significantly higher in PDV-inoculated leaves at 7 and 14 dpi (Figure 11).

## 4. Discussion

### 4.1. Symptoms of PDV Infection and Immunofluorescent Localization of P1 Protein Within a Context of PDV CP Distribution

There are two major ways to detect/identify PDV infection. First, DAS-ELISA is generally useful for natural hosts [24,25,26,27,28,29]. Second, biological inoculation of susceptible test plants can be done, including *Cucumis sativus* (especially cv. Wisconsin and cv. Polan), *Cucurbita maxima* (for example, cv. Buttercup), and *Nicotiana tabacum* cv. Samsun [3,11,12,24]. Here, PDV multiplies to higher levels, easily detectable by DAS-ELISA [6]. In our research we have started analyzing the PDV test plant quinoa (*C. quinoa*). This species, according to the Plant Virus Online Database [13], is generally considered to be unsusceptible to all isolates/strains of PDV, while other *Ilarviruses* are able to infect quinoa [3]. Quinoa is a commonly distributed weed, but is also cultivated as an alternative to cereals [30]. In this work we investigate the immunity-like reaction of quinoa to PDV by using CTCF, DAS-ELISA, and immunofluorescent localization of PDV as well as anatomical and ultrastructural observations. We demonstrate that PDV generates chlorotic lesions on inoculated leaves at 7 dpi, also previously reported by Fulton [11] and Kozieł et al. [8] for cucumber cotyledons or inoculated leaves of tobacco [10]. However, for the latter two hosts, infection symptoms were also observed on upper leaves at 14 dpi by Fulton [11] and Kozieł et al. [9]. Moreover, Kozieł et al. [9] also observed deformation of leaf blades resulting from PDV infection. The fact that PDV is absent from systemic leaves suggests that it cannot move systemically to other parts of the plant. Fulton [11] and Waterworth and Fulton [31] suggested that in the case of the *Bromoviridae* family (of which PDV is a member), the absence of symptoms above the inoculation point strongly implies a lack of systemic viral transport. Nevertheless, we were also aware of asymptomatic chronic PDV infection in some natural hosts. Indeed, immunofluorescent localization of P1 protein, CTCF analysis, and detection of PDV CP by using DAS-ELISA, as well as qPCR for *MP* gene, strongly indicate the ability of PDV to replicate in quinoa tissues. 

PDV CP accumulates to higher levels than other PDV proteins [1,2,7,32]. CP is engaged in “genome activation” needed at stages before viral RNA replication, but also is crucial for creation of PDV viral particles [33]. PDV is transported in the form of viral particles [8], so CP is also needed for this process. Immunofluorescent localization of PDV P1 epitope showed deposition inside palisade and spongy mesophyll (7 dpi) but not in phloem and xylem tissues in virus-inoculated quinoa leaves. P1 reached phloem after 14 dpi, but the fluorescent signal was less intense than at 7 dpi. Similar patterns of P1 distribution were observed in tobacco leaves [9]. Moreover, in mock-inoculated and upper quinoa leaves, no P1 was localized. However, our observations in PDV-infected tobacco [9] revealed stronger fluorescence from P1 than in inoculated quinoa leaves, although for quinoa, changes (deformations) in palisade and spongy mesophyll cells were more severe than in tobacco [9]. The presence of P1 protein is an efficient marker of ongoing PDV replication [8,9]. Similarly, the absence of P1 protein in upper leaves potentially supports our hypothesis about a blockage of systemic PDV transport. Moreover, CTCF levels of PDV also decreased in already virus-inoculated leaves during the time of infection.

The DAS-ELISA results clearly show that the relative level of PDV CP in quinoa plants significantly decreased over time. More interestingly, DAS-ELISA did not detect CP not only in leaves above inoculation but also in the stem. These results show that CP was unable to translocate through stem to/from inoculated and upper leaves. Pallas et al. [1,2,32] stated that levels of CP in *Ilarviruses* may serve as a parameter of virus quantity, building the viral capsids. If so, we believe we have demonstrated that the relative level of PDV decreases in quinoa plants.

Moreover, decreased normalized relative expression of *MP* gene in PDV-inoculated leaves and the lack of expression in organs above the inoculation point indicate that quinoa has an immunity-like reaction. Based on observations of Kozieł et al. [8] in PDV-infected cucumber, both CP and MP proteins are equally important in cell-to-cell transport. Therefore, changes in CP and MP expression levels indicate that PDV infection has less mobility in quinoa plants, which represents the first evidence of an immunity-like reaction against PDV in *Chenopodium quinoa* plants.

### 4.2. Anatomical and Ultrastructural Changes in PDV-Infected Quinoa Plants

We observed high levels of deformations in spongy mesophyll and parenchyma cells at 14 dpi and 20 dpi but only in inoculated leaves. This is a unique feature of quinoa plants that was not observed in any natural or test hosts [1,3,7,11,29]. At 14 dpi we also spotted invaginations of cell walls and necrosis in phloem and xylem parenchyma tissue inside major vascular bundles. Such necrosis in different vascular tissues due to viral infection has been frequently noted not only for PDV [9] but also for other plant viruses, e.g., *Potato virus Y* (PVY, *Potyviridae*) [34]. Interestingly, changes in PDV-inoculated leaves were more severe at some distance from major vascular bundles, including deformations in almost all structural tissues and numerous cell wall invaginations. Our results clearly indicate that the anatomical response to PDV did engage almost all leaf tissues in quinoa. The following TEM analysis revealed changes in chloroplast ultrastructure at 7 dpi also in PDV-inoculated leaves. The electron-dense substance, probably lipids, accumulated first on the surface of many palisade mesophyll chloroplasts, and at 14 dpi evaginations of chloroplast envelope, and invaginations of cell wall were spotted in spongy mesophyll cells; at 20 dpi the chloroplasts became completely disintegrated. The localized alterations in chloroplasts in the early stages of infection were similar between susceptible (for example, cucumber) [8] and “resistant” (*C. quinoa*) hosts. However, the appearance of these changes varied over time. In susceptible cucumber hosts, the first stage of chloroplast alteration was translucent regions in the stroma between thylakoids, which was not observed in “resistant” *C. quinoa*. It was quickly followed by disintegration of whole chloroplasts in cucumber leaves [8]. Moreover, the disintegration process of chloroplasts was much more intense in cucumber than in quinoa. This had a significant influence on the chlorosis level, which was greater in susceptible cucumber than in quinoa. Moreover, in cucumber, severe alteration of mesophyll chloroplasts was accompanied by changes of mitochondria, showing reduced cristae and large electron-translucent regions [8]. In quinoa plants, such changes have not been observed. The endoplasmic reticulum (ER) cisterns were also enlarged, especially in companion cells in the phloem. Alterations in chloroplasts and ER by PDV were observed in cucumber [8], with numerous electron-translucent regions [8] not observed in quinoa. Favali and Conti [35] found electron-translucent regions in bean chloroplasts infected with alfalfa mosaic virus (AMV). Such severe alterations in quinoa chloroplasts could be an effect of ROS generation [36]. One ROS involved in signal transduction during viral infection [29] is H_2_O_2_, and we observed the presence of H_2_O_2_ in changed quinoa chloroplasts. In resistant plants, ROS (also H_2_O_2_) is frequently associated with a hypersensitive response (HR) [36], which may induce high levels of cell deformation. As we mentioned above, cell deformation and high levels of H_2_O_2_ are also linked to PDV infection, so deformations in quinoa cells could be a possible result of H_2_O_2_ accumulation. This is supported by statistical analysis showing the level of H_2_O_2_ to increase over 92% (based on CTED) and 63% in spectrophotometric analysis.

As in tobacco and cucumber [8,9], we observed PDV VPs and MP epitope inside quinoa cytoplasm near plasmodesmata with extended SEL. Moreover, PDV VPs and MP epitope were found near potentially movement protein-induced tubular structures that pass through to the cell wall. Changes in plasmodesmata and generation of microtubules likely reflect the intercellular PDV transport [8] in this host, whereas the presence of vesicle pockets and spherules where P1 protein was localized via immunofluorescence serves as evidence of active replication of PDV, similar to other ilarviruses [9].

Ultrastructural analysis also reveals changes in vascular tissues such as phloem, where PDV was observed in both parenchyma and companion cells, likely involved in the production of PDV VPs. The resulting viral particles presence could cause necrosis of companion cells. This necrotization could be potentially responsible for blocking virus transport through sieve tubes. The presence of PDV in xylem parenchyma generated clearly visible sequential modification of xylem vessels, filled initially with electron-dense substance (possible/putative phenolic compounds), finally clogging the whole cells. As has been shown by Kozieł et al. [8,9], systemic movement of PDV in tobacco and cucumber is associated with phloem and xylem cells. If so, then absence of PDV and MP in quinoa leaves above the inoculation site (based on DAS-ELISA, immunofluorescence, and expression of *MP*) could be an effect of simultaneously combining three types of characteristic ultrastructural change that influence quinoa reaction to PDV. First, large-scale cell deformation potentially stalls intercellular transport to the vascular tissues. The second is necrotization of companion cells, possibly indicating that PDV is no longer able to undergo systemic transportation via sieve tubes. The last one is deposition of electron-dense substance in xylem vessels, which is likely to block systemic PDV transport. These elements taken together may contribute to the immunity-like reaction against PDV in quinoa. Observed ultrastructural changes in quinoa cells are also supported by high levels of H_2_O_2_, which may potentially increase the effectiveness of antiviral reactions. Moreover, not only the character of changes in xylem and phloem but also the rate of change could have an influence on quinoa resistance.

## 5. Conclusions

Our study combines immunolocalization of P1, analysis of immunofluorescence signal, statistical assessment of PDV-CP detection, and corrected total electron density assessment of H_2_O_2_ to better understand the reaction of *C. quinoa* to PDV. TEM ultrastructural analysis demonstrated several specific types of ultrastructural changes in quinoa associated with different types of vascular tissues, which could induce/retain PDV in the inoculation zone of the leaf. It was noticed that quinoa plants had symptoms of PDV inoculation. The localization of the main component of replication complex-P1 protein confirmed the potential and possible PDV replication process in inoculated leaves. Interestingly, the deposition of PDV P1 protein as well as virus amount was significantly decreased from 7 and 14 dpi in inoculated leaves. Moreover, the completely lack of the P1 protein deposition was noticed in leaves above inoculation point. Similar observations to P1 protein were detected in the case of PDV MP. The relative expression level of *MP* was detectable only in inoculated leaves but with 80% decrease tendency between 7 and 14 dpi. Moreover, immunolabeling of PDV MP confirmed deposition inside plasmodesmata and potential tubular structures. Therefore, between 7 and 14 dpi after inoculation, the virus could move through plasmodesmata. Despite these observations, PDV MP was not present in tissues above inoculation site, which was confirmed by relative expression of *MP*. Intensive necrotizations of companion cells, electron-dense substance inside xylem tracheary elements, and deformation of plant cells accompanied by decreased amount of virus with increased deposition of H_2_O_2_ influence the restriction of virus systemic translocation through vascular tissues. Because of such specific and quick reactions, the inoculum is eliminated from the leaf, and PDV is not transported systemically to other plant organs. DAS-ELISA and qPCR tests confirmed the absence of the virus in the stem and other leaves and the decreased level of virus in inoculated leaves. *C. quinoa* reactions strongly suggest the presence of some immunity-like reaction to PDV. Further studies based on molecular techniques are required to investigate more deeply the virus fitness and thus the resistance potential in *C. quinoa* plants. Likewise, quinoa–PDV interactions should be investigated in near the future from the point of view of genetic and/or molecular factors possibly engaged in the resistance response. These results could then serve as models for searching the types of antiviral resistance in other plants/crops.

## Supplementary Materials

The following are available online at https://www.mdpi.com/2073-4409/9/1/148/s1, Scheme S1, Tables S1–4, Figures S1A,B and S2. Scheme S1: Genome structure of PDV, Tables S1 and S2: Sequence of primers and qPCR conditions. Table S3: Characteristic of qPCR reaction. Table S4 and Figure S1A,B: PDV detection and relative virus concentration assessment using DAS-ELISA in quinoa leaves. Figure S2: Corrected total electron density (CTED) of cerium (IV) perhydroxide precipitate in mock- and PDV-inoculated quinoa leaves (7 and 14 dpi) combined with ANOVA.
