# Supplementary material for: Modifications in Tissue and Cell Ultrastructure as Elements of Immunity-Like Reaction in Chenopodium quinoa against Prune Dwarf Virus (PDV)
Source: Cells. 2020 Jan 8;9(1):148. doi: 10.3390/cells9010148 (PMC7017086; doi:10.3390/cells9010148)
Supplement: Supplementary file 1 [file cells-09-00148-s001.pdf]

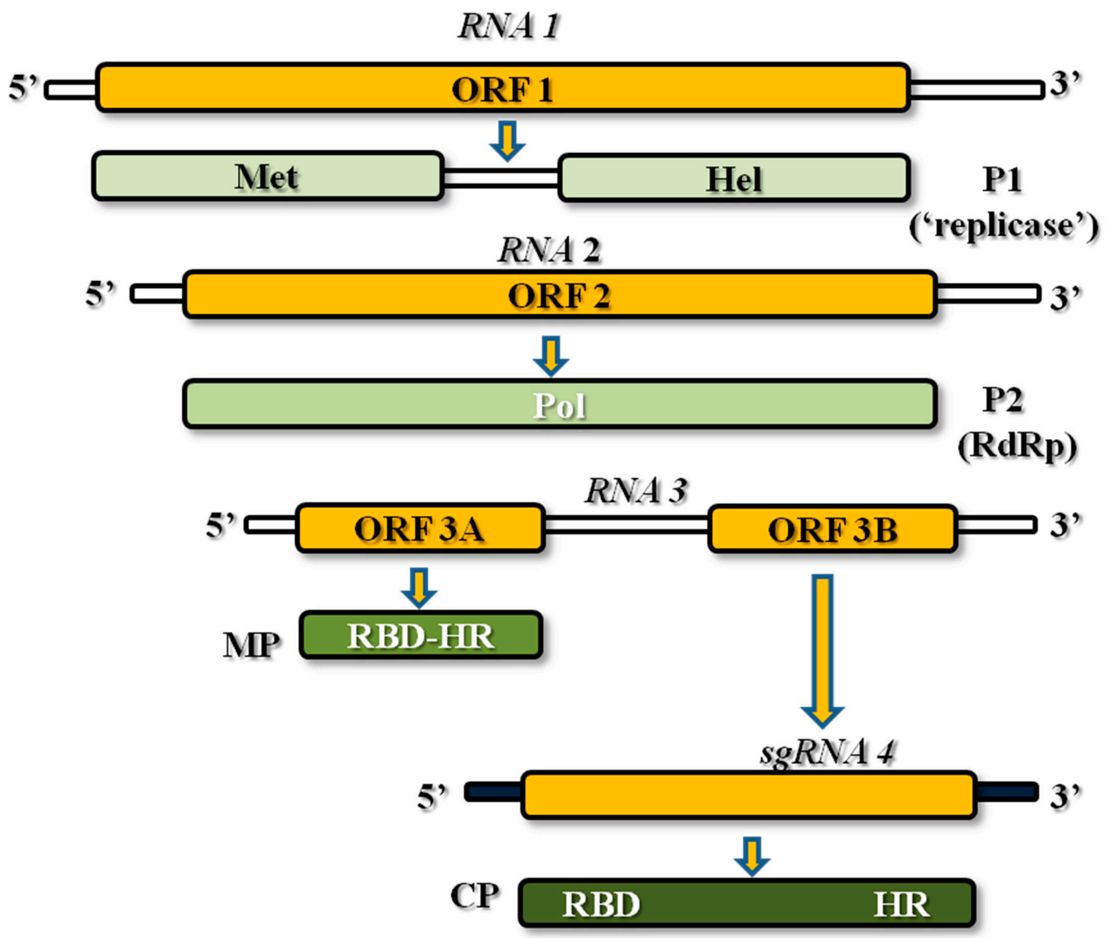

Scheme S1. Genome of *Prune dwarf virus* (PDV). Scheme presenting the ORFs and proteins encoded by PDV. Encoded proteins: P1 with methyltransferase and helicase domains, P2—polymerase, MP—movement protein, CP—coat protein. ORF—open reading frame, RBD—RNA binding domain, HR—hydrophobic region.

Table S1. Accessions of genes, sequences of primers, temperature of annealing, concentration in reaction, and product lengths for real-time qPCR. bp, base pair.

**Genes**	**Gene ID**	**Forward primer**	**Reverse primer**	**Temperature of primer annealing (°C)**	**Concentration in reaction (μM)**	**Product length (bp)**
Investigated						
MP	HM015770.1	GCTGACGATGGAGATGGAAT	CCAATTACCAAACGCGACTT	58	0,5	20
Reference						
Actin	LOC110724665	AGGAGGGTCTATCCTTGCGT	TGAACAATTGATGGGCCGGA	58	0,5	180

Table S2. Conditions of qPCR reaction of investigated *MP* gene and also for reference. Fluorescence signal reading set on the end of stage marked by asterisk (*).

Program	***Parameters***
Preliminary denaturation	95 °C for 5 min
Amplification (35 cycles)	95 °C for 10 s<br>58 °C for 10 s<br>72 °C for 20 s*
Melting curve	65–95 °C; 0.1 °C/s

Table S3. Characteristic of qPCR reaction for individual pairs of primers.

Gene	***Reaction efficency [%]***	***R^2 factor***
Actin	92,5	0,999
MP	99,4	0,998

Table S4. PDV detection assessment using double antibody sandwich enzyme-linked immunosorbent assay (DAS-ELISA) on quinoa leaves. Mean optical density (OD_{405nm}) values are presented with standard deviations (±s). Presence of PDV (+) is confirmed in samples with mean OD_{405nm} above 0.200.

**Sample**	**Mean OD_{450nm} ±s**	**Result of test: presence (+) or absence (–) of PDV**
Buffer	0.0001 ± 0.002	–
Mock-inoculated quinoa leaves (7 dpi)	0.0101 ± 0.02	–
Stem between mock-inoculated point and leaves above (7 dpi)	0.0110 ± 0.01	–
Leaves above mock-inoculation point (7 dpi)	0.0101 ± 0.02	–
PDV-inoculated quinoa leaves (7 dpi)	**0.5600 ± 0.03**	**+**
Stem between PDV-inoculation point and leaves above (7 dpi)	0.0190 ± 0.001	–
Leaves above PDV inoculation (7 dpi)	0.0110 ± 0.01	–
Mock-inoculated quinoa leaves (14 dpi)	0.0120 ± 0.02	–
Stem between mock-inoculated point and leaves above (14 dpi)	0.0123 ± 0.01	–
Leaves above mock-inoculation point (14 dpi)	0.0120 ± 0.02	–
PDV-inoculated quinoa leaves (14 dpi)	**0.250 ± 0.01**	**+**
Stem between PDV inoculation point and leaves above (14 dpi)	0.017 ± 0.02	–
Leaves above PDV inoculation (14 dpi)	0.012 ± 0.01	–
Leaves from mock-inoculated quinoa (20 dpi)	0.011 ± 0.02	–
Stem between mock-inoculated point and leaves above (20 dpi)	0.013 ± 0.01	–
Stem between PDV inoculation point and leaves above (20 dpi)	0.017 ± 0.02	–
Leaves above PDV inoculation (20 dpi)	0.011 ± 0.02	–

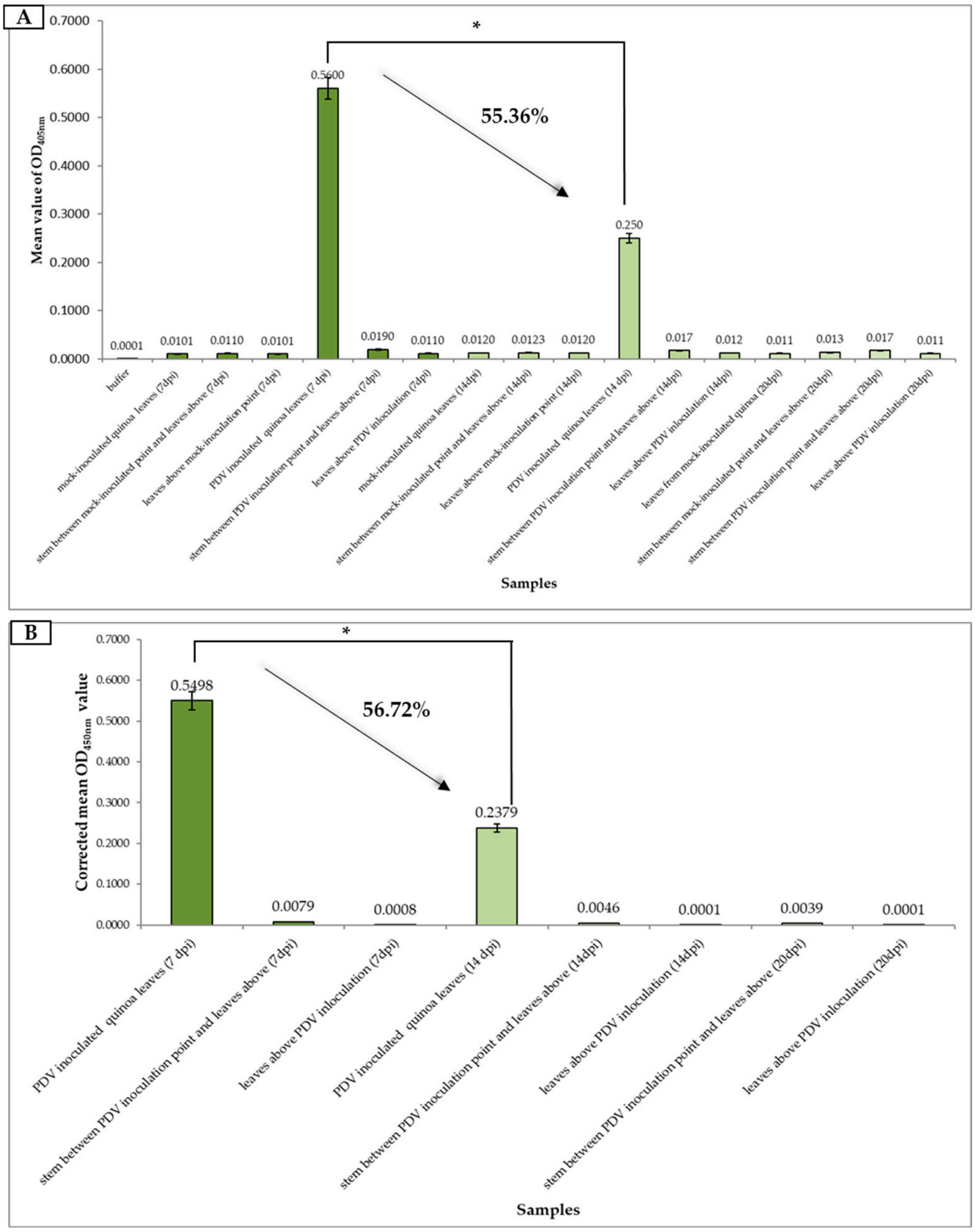

Figure S1. PDV detection and relative virus concentration assessment by using DAS-ELISA in quinoa leaves. The mean of OD_{405nm} and corrected OD_{405nm} values were assessed with ANOVA statistics method. (A) The assessment of the mean OD_{405nm}. Black arrows show the per cent decrease in PDV relative concentration during infection process. (B) The assessment of the corrected mean OD_{405nm} (mean OD_{405nm} of sample from PDV inoculated plant- sum of means OD_{405nm} of buffer and appropriate mock inoculated plant). Black arrows show the per cent decrease in PDV relative concentration during infection process in the inoculated leaves. Mean values of OD_{405nm} corrected OD_{405nm} were estimated at the $p < 0.05$ level of significance using post-hoc Tukey HSD test. The statistical significant values on (A) and (B) are marked by (*).

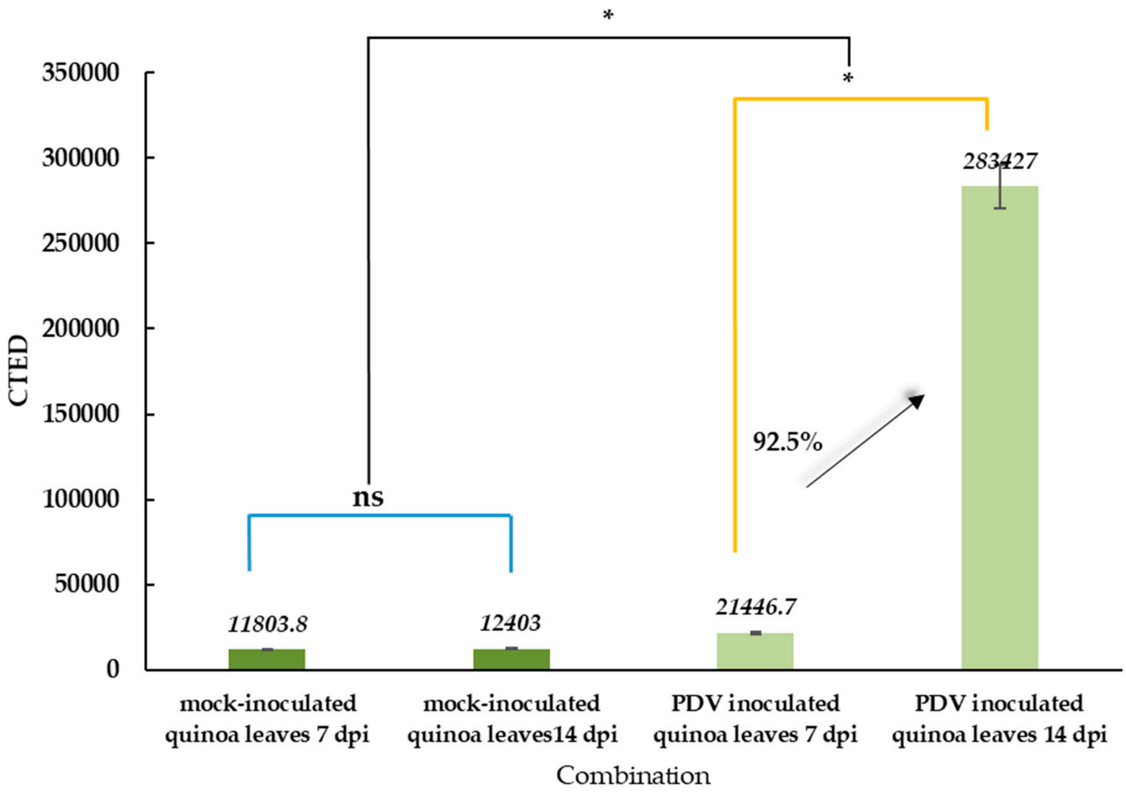

Figure S2. Corrected total electron density (CTED) of cerium (IV) perhydroxide precipitate in mock- and PDV-inoculated quinoa leaves (7 and 14 dpi) combined with ANOVA. Black arrow depicts percentage increase in hydrogen peroxide amount over the time of infection in PDV-inoculated leaves. Mean CTED values were evaluated at the $p < 0.05$ level of significance using Tukey's post hoc HSD test. Nonsignificant (ns) values within blue bracket. Significant statistical values between PDV-inoculated quinoa leaves at 7 and 14 dpi within orange bracket (*). Significant values between PDV- and mock-inoculated quinoa leaves at 7 and 14 dpi marked by black bracket with asterisk (*).
